# Copolymerization of Ethylene with Functionalized 1,1-Disubstituted Olefins Using a Fluorenylamido-Ligated Titanium Catalyst

**DOI:** 10.3390/polym16020236

**Published:** 2024-01-15

**Authors:** Oluwaseyi Aderemi Ajala, Moeko Ono, Yuushou Nakayama, Ryo Tanaka, Takeshi Shiono

**Affiliations:** Graduate School of Advanced Science and Engineering, Hiroshima University, Kagamiyama 1-4-1, Higashihiroshima 739-8527, Japan; d214279@hiroshima-u.ac.jp (O.A.A.); yuushou@hiroshima-u.ac.jp (Y.N.); rytanaka@hiroshima-u.ac.jp (R.T.)

**Keywords:** functional polyolefins, coordination polymerization, copolymerization, titanium catalyst

## Abstract

Considering the sustainability of material development, coordination polymerization catalysts effective for 1,1-disubstituted olefins are receiving a great deal of attention because they can introduce a variety of plant-derived comonomers, such as β-pinene and limonene, into polyolefins. However, due to their sterically encumbered property, incorporating these monomers is difficult. Herein, we succeeded in the copolymerization of ethylene with various hydroxy- or siloxy-substituted vinylidenes using a fluorenylamido-ligated titanium catalyst–MMAO system. This is the first example of ethylene/polar 1,1-disubstituted olefins’ copolymerization using an early transition metal catalyst system. The polymerization proceeded at room temperature without pressurizing ethylene, and high-molecular-weight, functionalized polyethylene was obtained. The obtained copolymer showed a reduced water contact angle compared with that of the ethylene/isobutene copolymer, demonstrating the increment in hydrophilicity by hydroxy groups.

## 1. Introduction

Olefin polymerization and copolymerization mediated by single-site transition metal catalysts have witnessed tremendous progress in the past 40 years, both in academia and industry, due to the discovery of methylaluminoxane (MAO), an excellent cocatalyst, by Kaminsky and Sinn [1]. Early transition metal complexes such as metallocenes have been the representative catalyst precursors, activated with MAO or borate cocatalysts to generate well-defined active species. Various copolymers with uniform comonomer distribution can be synthesized from the well-defined active species.

The incorporation of polar functional groups into polyolefin can considerably improve their surface characteristics like adhesion, dyeability, printability, and compatibility, and broaden their range of applications as commodity plastics. Therefore, in recent decades, the coordination copolymerization of ethylene and polar vinyl monomers has been vigorously investigated [2,3,4]. Late transition metal catalysts have played a leading role in this field because they are poisoned to a lesser degree by the electron-donating functional groups. Early transition metal catalysts can also be applied to comonomers with remote or protected functional groups [5]. Some group 4 metal catalysts are shown to be tolerant to polar functional groups like bulky amines without the aid of alkylaluminum [6]. By using these catalyst systems, high-molecular-weight crystalline polyolefins with a small number of functional sidechains containing carbonyl, amino, and hydroxy groups can be synthesized.

Recently, the use of 1,1-disubstituted olefins in coordination polymerization is drawing much attention in view of the sustainability of material development because many plant-derived 1,1-disubstituted olefins, such as pinene and limonene, are available. Incorporating these comonomers has been challenging because of their sterically encumbered property. For example, ethylene (E)/isobutene (IB) copolymerization using bis(indenyl)ethylidene zirconium dichloride activated with MAO produces a low-molecular-weight copolymer with low IB content, even under the excess feed of IB to E [7]. Waymouth explored the cyclopolymerization of 2-methyl-1,5-hexadiene using a metallocene catalyst, and found that the vinyl groups inserted preferentially over the vinylidene groups, producing a regioregular polymer [8]. However, these examples show that the insertion of 1,1-disubstituted olefins into group 4 metal–carbon bond is essentially possible. 

**Figure 1:**
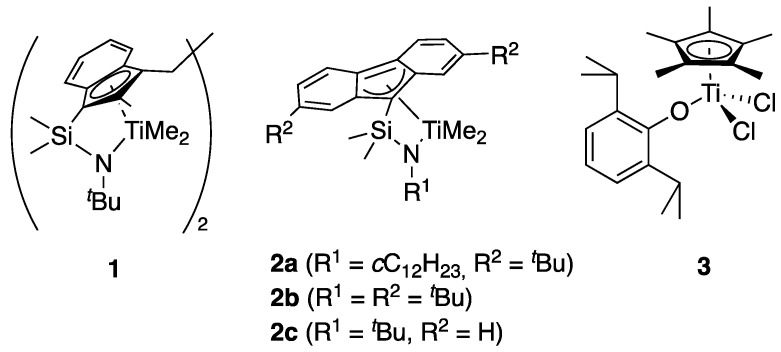
Effective early transition metal catalysts **1**–**3** for the copolymerization of ethylene and 1,1-disubstituted olefins.

Generally, constrained geometry catalysts (CGC) or modified half-titanocenes, which have open coordination sites for comonomers, are known to be effective for ethylene and non-polar 1,1-disubstituted monomers because they can accept various sterically encumbered monomers. The first example of an E/IB alternating copolymer was prepared by a CGC titanium catalyst with a cyclododecyl substituent on its nitrogen atom activated by a borate activator [9]. Marks showed that a binuclear CGC-type catalyst (**1**, Figure 1) effectively copolymerized ethylene and various isoalkenes [10,11]. We have demonstrated that the application of fluorenylamido-ligated titanium complex **2a**–**2c** can promote the copolymerization of ethylene with IB or limonene [12]. Recently, Nomura reported the copolymerization of ethylene and various plant-derived olefins such as limonene and β-pinene using half-titanocene complexes bearing a phenoxide ancillary donor, as represented by complex **3**, activated by MAO to produce a comonomer content of up to 3.6 mol% under a pressurized condition [13]. These catalysts show a high activity for the copolymerization of ethylene and other multi-substituted olefins such as 2-methyl-1-pentene (2M1P) [14,15] and 4-methylcyclopentene [16]. However, no examples of copolymerizing polar 1,1-disubstituted monomers using early transition metal catalysts have been reported. Late transition metal catalysts have made remarkable progress in the copolymerization of ethylene and polar 1,1-disubstituted monomers like methacrylates, reflecting their lower oxophilicity [17,18,19]. However, all these systems require high temperature (>95 °C) and/or ethylene pressure.

Here, we have investigated the copolymerization of ethylene with 1,1-disubstituted olefins, bearing hydroxy groups or its derivatives, using complex **2a**–**2c** activated with modified methylaluminoxane (MMAO). Although the hydroxy groups should be protected with silyl groups to achieve a high activity, the copolymerization proceeded at ambient pressure and temperature, producing high-molecular-weight (*M*_n_ > 10^5^), functionalized polyethylene. The siloxy groups can be deprotected with the standard procedure using tetrabutylammonium fluoride, and the obtained hydroxy-substituted polyethylene showed superior surface wettability than the polyethylene that possessed no polar functional groups.

## 2. Materials and Methods

All operations were performed under a nitrogen atmosphere using standard Schlenk techniques. Dry toluene, dichloromethane, and THF were purchased from Kanto Chemical Co. Inc. and used after passing through solvent purification columns. Titanium complex **2a**–**2c** [12], 6-methylhept-6-en-1-ol (**4a**) [20], and (+)-*trans*-β-terpineol (**6a**) [21] were synthesized according to the literature. Isoprenol (**5a**; TCI Chemicals), isobutene (TCI Chemicals, 9 wt% hexane solution), and 2-methyl-1-pentene (TCI Chemicals) were used as received. Modified methylaluminoxane (MMAO) was generously donated from Tosoh-Finechem Co., Ltd. as a 2.0 mol/L toluene solution. Other materials were used as received. 

NMR spectra were recorded on a Varian System 500 or a JEOL Lambda500 spectrometer at room temperature or 130 °C. The obtained spectra of ^1^H NMR and ^13^C NMR were referenced to the signal of a residual trace of the partially protonated solvents (^1^H: δ = 5.91 ppm (C_2_HDCl_4_) and 7.26 ppm (CHCl_3_)) and the signal of the solvent (^13^C: δ = 74.7 ppm (C_2_D_2_Cl_4_) and 77.1 ppm (CDCl_3_)), respectively. The high-resolution mass spectrometry of the new compounds was performed on a JEOL JMS-T100GCV spectrometer. The absolute molecular weights of polymers were determined on a Malvern HT350GPC chromatograph (*T* = 130 °C; eluent, *o*-dichlorobenzene) equipped with RI/light scattering/viscometer triple detectors. Differential scanning calorimetry (DSC) measurements were performed on a SHIMADZU DSC-60 system with a temperature elevation rate of 10 °C min^−1^. The water contact angle was measured by a KYOWA DM-300 contact angle meter using a half-angle method.

**Synthesis of Comonomer 4b**

In a 100 mL two-necked flask, imidazole (5.3 mmol, 0.36 g) and 6-methylhept-6-en-1-ol (**4a**, 2.1 mmol, 0.27 g) were charged under nitrogen and diluted with dichloromethane (3 mL). The solution was cooled to 0 °C, and *^i^*Pr_3_SiCl (2.5 mmol, 0.54 mL) was added dropwise. The mixture was warmed to room temperature and stirred overnight. The resulting solution was washed twice with water and once with brine, and the aqueous phase was extracted with dichloromethane (5 mL × 3). The combined organic phase was dried over MgSO_4_ and concentrated. The obtained crude product was purified with a silica gel column chromatography (5% EtOAc/hexane, *R*_f_ = 0.55), producing **4b** as a clear oil (0.35 g, 59%). ^1^H NMR (500 MHz, CDCl_3_): δ: 4.69–4.67 (m, 1H), 4.66–4.64 (m, 1H), 3.67 (t, ^3^*J*_HH_ = 7 Hz, 2H), 2.01 (t, ^3^*J*_HH_ = 8 Hz, 2H), 1.71 (s, 3H), 1.59–1.51 (m, 2H), 1.48–1.41 (m, 2H), 1.38–1.30 (m, 2H), 1.13–1.03 (m, 21H). ^13^C NMR (125 MHz, CDCl_3_) δ: 146.3, 109.8, 63.6, 38.0, 33.1, 27.6, 25.7, 22.5, 18.2, 12.2. GC/FI-MS, Calcd for C_17_H_36_OSi: 284.25354 [M^+^·], Found: 284.25315.

**Synthesis of Comonomer 5b**

In a 100 mL two-necked flask, imidazole (75 mmol, 5.1 g) and isoprenol (**5a**, 30 mmol, 3.04 mL) were charged under nitrogen and diluted with dichloromethane (30 mL). The solution was cooled to 0 °C, and *^i^*Pr_3_SiCl (36 mmol, 7.6 mL) was added dropwise. The mixture was warmed to room temperature and stirred for 16 h. The resulting solution was washed twice with 10 mL of aqueous saturated NaHCO_3_. The collected aqueous phase was extracted with dichloromethane (5 mL × 3). The combined organic phase was dried with MgSO_4_ and filtered, and the solvent was evaporated. The obtained crude product was purified with a silica gel column chromatography (hexane, *R*_f_ = 0.30), producing **5b** as a clear oil (7.20 g, >99%).

1H NMR (in CDCl_3_, 400 MHz), *δ*: 4.76 (1H, m), 4.71 (1H, m), 3.79 (2H, t, ^3^*J*_H-H_ = 7 Hz), 2.28 (2H, t, ^3^*J*_H-H_ = 7 Hz), 1.76 (3H, s), 1.15–1.00 (21H, m). ^13^C NMR (in CDCl_3_, 100 MHz), *δ*: 143.3, 111.6, 62.6, 41.4, 23.1, 18.2, 12.2 (^1^*J*_Si-H_ = 58 Hz). GC/FI-MS, Calcd for C_14_H_30_OSi: 242.20659 [M^+^·], Found: 242.20654.

**Synthesis of Comonomer 6b**

In a 100 mL two-necked flask, (+)-*trans*-β-terpineol (**6a**, 2.0 mmol, 309 mg) and 2,6-lutidine (2.0 mmol, 0.23 mL) were charged under nitrogen and diluted with dichloromethane (3 mL). The solution was cooled to 0 °C, and *^i^*Pr_3_SiOTf (2.0 mmol, 0.54 mL) was added dropwise. The mixture was warmed to room temperature and stirred for 6 h. The resulting solution was washed three times with 5 mL of aqueous saturated NaHCO_3_ solution. The collected aqueous phase was extracted with dichloromethane (3 mL × 3). The combined organic phase was dried with MgSO_4_ and filtered, and the solvent was evaporated. The obtained crude product was purified with a silica gel column chromatography (hexane, *R*_f_ = 0.79), producing **6b** as a clear oil (282 mg, 45%). 

1H NMR (in CDCl_3_, 400 MHz), *δ*: 4.70 (brm, 1H), 4.67 (brm, 1H), 1.84–1.07 (36H, m). ^13^C NMR (in CDCl_3_, 100 MHz), *δ*:151.3, 108.2, 71.4, 45.2, 40.4, 31.1, 27.2, 21.0, 18.7, 13.7. [α]_16_^D^ = 3.4° (*c* = 1.9, in CHCl_3_), GC/FI-MS, Calcd for C_19_H_38_OSi: 310.26919 [M^+^·], Found: 310.26954.

**Copolymerization of ethylene and polar 1,1-disubstituted olefins with semi-batch process**

A representative procedure for Table 1, Run 3, is described here. In a 100 mL two-necked flask, **4b** (2.46 mmol, 597 mg) was weighed under nitrogen and dissolved in 6 mL of toluene. To this solution, toluene solutions of modified methylaluminoxane (MMAO) (2.0 M, 2.0 mL, 4.0 mmol) and 2,6-di-*tert*-butyl-4-methylphenol (BHT, 0.30 M, 1.0 mL, 0.30 mmol) were added. The nitrogen in the headspace of the flask was removed under vacuum, and ethylene was introduced back at ambient pressure until saturation. Polymerization was started by adding a solution of titanium complex **2a** (20 μmol, 11.9 mg in 1.0 mL toluene), and the reaction mixture was magnetically stirred for 20 min under a flow of ethylene. The polymerization was terminated by adding 2 mL of MeOH. The resulting mixture was poured into 200 mL of MeOH containing 4 mL of concentrated HCl to separate the polymer and catalyst. The precipitated polymer was collected by filtration and dried for 4 h under vacuum at 60 °C to obtain a constant weight. A total quantity of 363 mg of colorless polymer was obtained.

**Copolymerization of ethylene and silyl-protected polar 1,1-disubstituted olefins with batch process**

A representative procedure for Table 2, Run 9 was described here. To a 300-mL two-necked flask, comonomer **5b** (2.46 mmol, 699 mg) was weighed, and MMAO (8.0 mmol in 3.7 mL toluene) was added. The nitrogen in the headspace of the flask was removed under vacuum, and 340 mL of ethylene (13.9 mmol at 25 °C) was introduced back. Polymerization was started by adding a solution of titanium complex **2b** (20 μmol, 9.7 mg in 1 mL toluene), and the flask was sealed. The reaction mixture was magnetically stirred for 60 min maintaining 25 °C. The polymerization was terminated by adding 2 mL MeOH. The resulting mixture was poured into 100 mL of MeOH containing 10 mL of concentrated HCl to separate the polymer and catalyst. The precipitated polymer was collected by filtration and dried for 4 h under vacuum at 60 °C to obtain a constant weight. 118 mg of colorless polymer was obtained.

**Deprotection of silyl groups in the copolymer**

In a Schlenk tube, ethylene/**4b** copolymer (40 mg, **4b** content = 84 µmol) obtained in Table 1, Run 3, was dissolved in chlorobenzene (2.0 mL) at 100 °C. To this solution, 0.10 mL of tetrabutylammonium fluoride solution (TBAF, 1.0 M in THF, 0.20 mmol) was added, and the mixture was stirred at 100 °C for 15 h. The polymer was precipitated by adding excess methanol and collected. The obtained polymer was dried under vacuum at 60 °C for 6 h, producing 16 mg (quant.) of the deprotected copolymer.

**Preparation of polymer films for water contact angle measurement**

Before the sample preparation, polymer was dissolved in chlorobenzene, filtered through a 0.045 mm stainless mesh, and reprecipitated in methanol to remove impurities. A thin film was prepared by pressing the polymer at 180 °C and 4.0 MPa for 5 min. Seven measurements at different positions of the film were averaged to determine the water contact angle. 

## 3. Results and Discussion

**Scheme 1:**
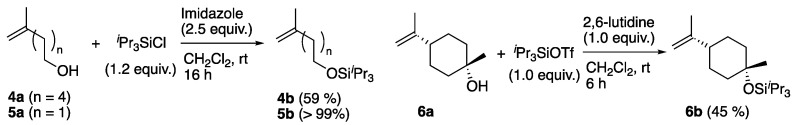
Synthesis of silyl-protected polar 1,1-disubstituted olefins **4b**–**6b**.

Previously, we have succeeded in copolymerizing ω-hydroxyalkenes using fluorenylamido-ligated titanium complex by pretreating the comonomer with triisobutylaluminium before polymerization to prevent catalyst poisoning by the hydroxyl functional group [22]. Converting the hydroxy group to silyl ether is also a conventional way for the efficient homo- and copolymerization of ω-hydroxyalkenes, and bulky trialkylsilyl groups tend to show a high polymerization activity [23]. According to these previous studies, 1,1-disubstituted olefins with siloxy groups (**4b**–**6b**) were synthesized and applied for the copolymerization. The conversion of alcohols **4a**–**6a** to silyl ether **4b**–**6b** was performed using *^i^*Pr_3_SiCl/imidazole (for primary alcohols) or *^i^*Pr_3_SiOTf/2,6-lutidine (for a tertiary alcohol) conditions (Scheme 1). A large-scale reaction was possible for the conversion of isoprenol (**5a**), producing 7.2 g of silyl ether **5b** in an almost quantitative manner. The obtained comonomer was purified with silica gel column chromatography prior to its application in the copolymerization process. 

**Scheme 2:**
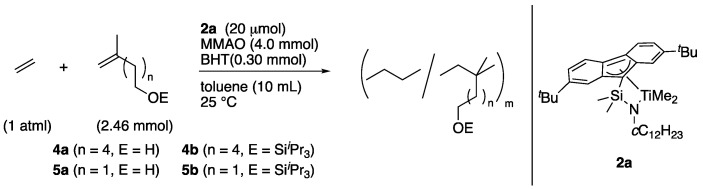
Copolymerization of ethylene and functionalized 1,1-disubstituted olefins **4** and **5** using **2a**-MMAO/BHT system.

First, the copolymerization of ethylene and 6-methyl-hept-6-en-1-ol derivatives (**4b**) was performed in a semi-batch process using **2a**-MMAO/BHT system (Scheme 2, Table 1). Here, BHT is added to modify the residual trialkylaluminums, which can competitively coordinate to the metal center of the catalyst with monomers and reduce the polymerization activity [24]. Although the polymer yield was lower than that of the copolymerization with unfunctionalized 1,1-disubstituted olefins such as isobutene and 2-methyl-1-pentene (Run 1, 2), high-molecular-weight copolymer was obtained with longer polymerization time at ambient temperature and pressure (Run 3). The direct copolymerization of unprotected **4a** (run 4) also proceeded when the amount of MMAO was increased, probably because an excess amount of alkylaluminum can mask the hydroxy group of **4a**.

**Table 1:** Copolymerization of ethylene and 1,1-disubstituted olefins using **2a**-MMAO/BHT system ^a^.

Run	Comonomer	Time (min)	Yield (mg)	*M*_n_ ^b^ (10^4^)	*Ɖ* ^b^	*T*_m_ ^c^ (°C)	Δ*H* ^c^ (J/g)	Comonomer Content ^d^ (mol%)
1	Isobutene	10	249	11	2.6	128	50	10.9
2	2M1P	10	413	13	1.9	126	13	8.7
3	**4b**	20	363	23	4.5	130	39	6.0
4 ^e^	**4a**	20	40	48	2.0	133	32	1.8
5	**5b**	20	35	99	1.4	133	76	0.9
6 ^f^	**5b**	20	43	0.6	14.6	127	58	2.4
7 ^e^	**5a**	20	16	50	1.8	135	126	- ^g^

^a^ Polymerization conditions: [**2a**] = 20 μmol, [Comonomer] = 2.46 mmol, [MMAO] = 4.0 mmol, [BHT] = 0.30 mmol, [C_2_H_4_] = 1 atm (semi-batch), toluene = 10 mL, temp = 25 °C. ^b^ Absolute molecular weight measured by GPC equipping RI-light scattering-viscometer triple detectors. ^c^ Determined by DSC. ^d^ Determined by ^1^H NMR. ^e^ [MMAO] = 8.0 mmol. ^f^ Temp. = 80 °C. ^g^ Not detected.

**Figure 2:**
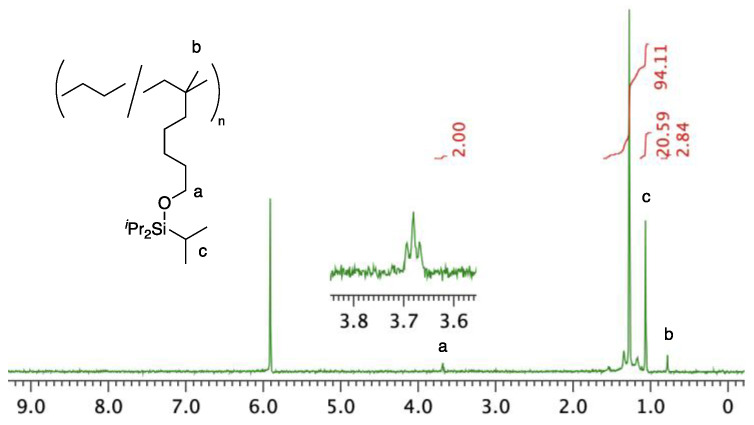
^1^H NMR spectrum of ethylene/**4b** copolymer obtained in Table 1, Run 3 (500 MHz, in C_2_D_2_Cl_4_, 130 °C).

**Scheme 3:**
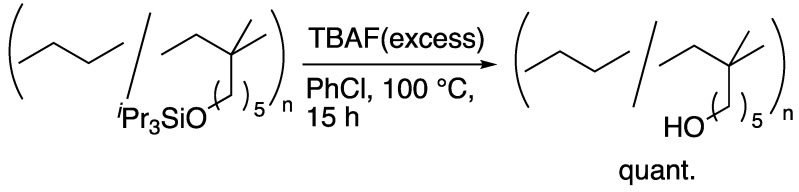
Deprotection of silyl groups in ethylene/**4b** copolymer.

The analysis of an ethylene/**4b** copolymer using ^1^H NMR spectrum showed a triplet signal at 3.68 ppm, which is assigned to methylene protons adjacent to the oxygen atom, indicating the incorporation of **4b** (Figure 2). Furthermore, a singlet 3H signal at 0.78 ppm and a 21H signal at 1.05 ppm are assigned to terminal methyl and isopropylsilyl protons, respectively. The methylene signal appears at the different chemical shift (3.53 ppm) in the ethylene/**4a** copolymer (Figure S9), indicating that the silyl ether remained in the copolymer. Thus, the comonomer content was calculated from the integral ratio of the -CH_2_OH signals and signals in the aliphatic region. A further deprotection of silyl groups can be performed by heating the polymer solution in chlorobenzene with excess tetrabutylammonium fluoride (TBAF). The reaction proceeded quantitatively, and hydroxy-substituted polyethylene was recovered (Scheme 3). The deprotection of silyl group was confirmed by a higher field shifted methylene signal in the ^1^H NMR spectrum (Figure S10). Moreover, the IR spectrum of the deprotected copolymer showed a broad signal around 3300 cm^−1^, which is not observed in the ethylene/**4b** copolymer, showing the presence of hydroxy groups generated via the elimination of silyl groups (Figure S15).

**Figure 3:**
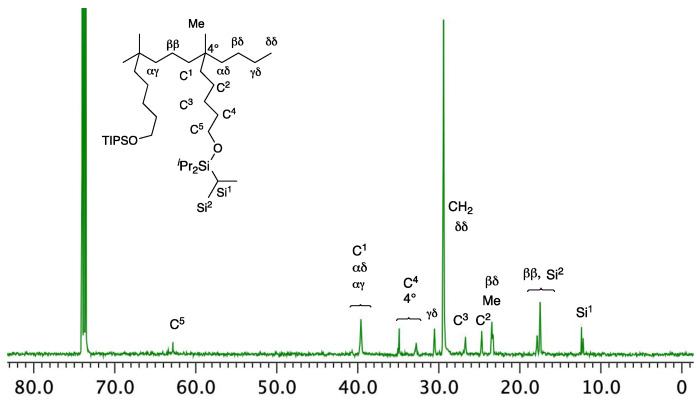
^13^C NMR spectrum of E/**4b** copolymer obtained in Table 1, Run 3 (125 MHz, in C_2_D_2_Cl_4_, 130 °C).

The ^13^C NMR signals of the copolymer were assigned according to the previous assignment of the copolymer of ethylene and various 1,1-disubstituted olefins [12,13,14,15,18,19]. In the ^13^C NMR spectrum of the ethylene/**4b** copolymer, signals at 40 and 65 ppm, which are assigned to the isolate branching of the polyethylene chain and carbon adjacent to the oxygen atom, are observed, suggesting the isolated structure of the **4b** unit (Figure 3). Successive insertion of **4b** would not occur because no signals were observed between 60 and 55 ppm, where a carbon signal of polyisobutene appears. The observed sole melting temperature (*T*_m_, 130 °C) is lower than that of homopolyethylene but high for the 6.0 mol% of incorporation. The bimodal GPC trace of the copolymer indicated that a low-molecular-weight fraction with high **4b** incorporation exists. A separation of these fractions using extraction or crystallization in xylene was not possible, probably because the average molecular weight of the lower *M*_w_ fraction still exceeds 10^5^ (Figure S14). 

**Figure 4:**
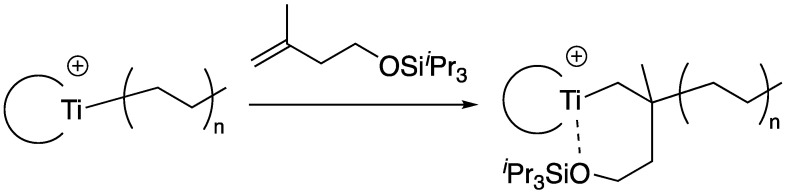
Structure of active species after the insertion of comonomer **5b**.

Comonomer **5b**, which has a shorter distance between C=C double bond and protected hydroxy group than **4b**, was also copolymerized with ethylene, although the polymer yield was much lower than the ethylene/**4b** copolymerization (Run 5). Polymerization in the heated condition largely decreased the molecular weight of the polymer, but a higher comonomer incorporation than polymerization at room temperature was achieved, probably because ethylene concentration in the reaction medium was lowered by heating (run 6). The direct copolymerization of isoprenol (**5a**) was unsuccessful, typically yielding a small amount of homopolyethylene (Run 7). In the copolymerization or homopolymerization of hydroxy- or siloxy-substituted vinyl monomers using group 4 metal catalysts, monomers with shorter distance between C=C double bond and functional groups tend to show a lower activity and a lower incorporation ratio, probably because of the bulkiness of the substituent [25,26,27]. Furthermore, **5b** would reduce the activity after the insertion into propagating chain end because oxygen atom of **5b** can easily interact with titanium center via a six-membered ring formation, which prevents the further coordination of monomers (Figure 4). 

Our previous research shows several substituent effects of fluorenylamido-ligated titanium complexes on various olefin polymerizations [12]. Generally, introducing electron-donating alkyl groups on the fluorenyl 2,7-position greatly improves the activity. The incorporation ratio of bulky comonomers is enhanced when tertiary alkyl group on the nitrogen was replaced with less bulky secondary alkyl group. Based on these previous results, the behavior of catalysts **2b** and **2c** in the copolymerization of ethylene and **5b** was compared with catalyst **2a** (Table 2). Here, the copolymerizations are conducted without the addition of BHT, and polymers with broader molecular weight distributions were obtained. The multimodal distribution of molecular weight may suggest the presence of multiple active species differentiated by the coordination of residual trialkylaluminums. Copolymerization using non-substituted fluorenylamido-ligated titanium catalyst **2c** was unsuccessful and produced only a small amount of polymer (Run 10), whereas copolymerization using catalyst **2b** proceeded with a higher activity than **2a** with a lower incorporation ratio of **5b** (Run 8 vs. Run 9). These polymerization behaviors can be explained by the previously reported tendency of other polymerizations using **2a**–**2c**. In the ^1^H NMR spectrum of the copolymer obtained from Run 9, two signals are observed at 3.5–3.7 ppm, which is assigned to methylene groups (Figure S11). These two signals indicate that the silyl ether was removed during the polymerization, and the obtained E/**5b** copolymer possesses unprotected hydroxy groups.

**Table 2:** Catalyst effect on the copolymerization of ethylene and **5b** ^a^.

Run	Catalyst	Yield (mg)	*M*_n_ ^b^ (10^4^)	*Ɖ* ^b^	*T*_m_ ^c^ (°C)	Δ*H* ^c^ (J/g)	Comonomer Content ^d^ (mol%)
8	**2a**	18	6.7	33.7	130	39	2.8
9	**2b**	118	2.8	6.0	126	13	1.1
10	**2c**	7	1.9	9.9	- ^e^	- ^e^	- ^e^

^a^ Polymerization conditions: [Ti] = 20 μmol, [**5b**] = 2.46 mmol, [MMAO] = 8.0 mmol, [C_2_H_4_] = 13.9 mmol (batch), toluene = 5.3 mL, time = 60 min, and temp = 25 °C. ^b^ Absolute molecular weight measured using GPC equipped with RI-light scattering-viscometer triple detectors. ^c^ Determined using DSC. ^d^ Determined using ^1^H NMR. ^e^ Not determined because of very small yield.

**Scheme 4:**
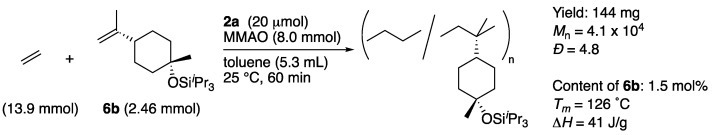
Copolymerization of ethylene and **6b**.

**Figure 5:**
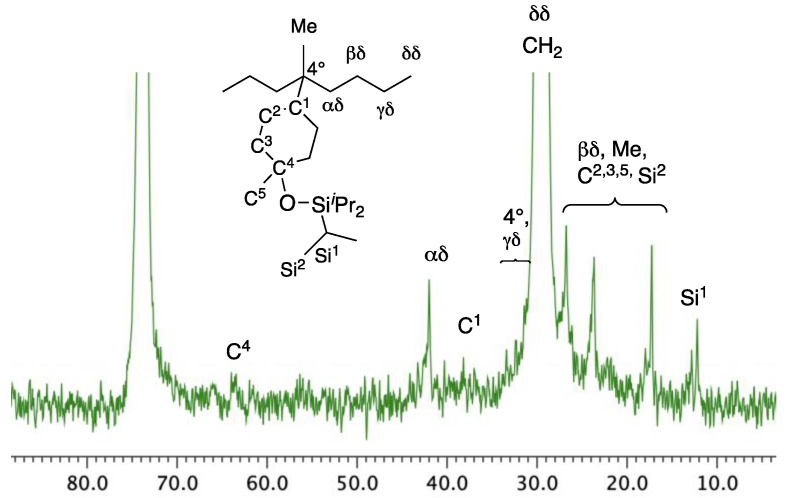
^13^C NMR spectrum of E/**6b** copolymer (125 MHz, in C_2_D_2_Cl_4_, 130 °C).

A plant-derived monomer **6b** can also be incorporated into polyethylene in a batch polymerization process with a higher concentration of **6b** (Scheme 4). The incorporation ratio of **6b** was calculated using ^13^C NMR because there is no methylene adjacent to the hydroxy group. By comparing the integral ratio of signals at 43 ppm (αδ) and 30 ppm (CH_2_ in methylene unit, Figure 5), the comonomer content was calculated to be 1.5 mol%. Silyl groups on the hydroxy groups would not completely be deprotected because carbons assigned to isopropylsilyl groups were observed at 12–14 ppm.

**Figure 6:**
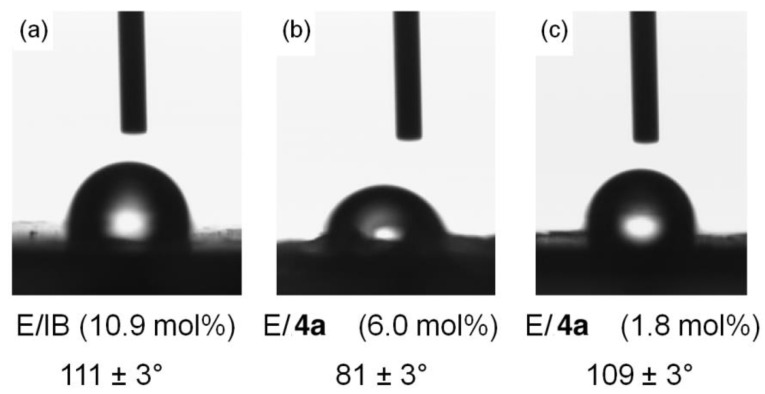
Comparison of water contact angle on (**a**) ethylene/isobutene (incorp. = 10.9 mol%), (**b**) ethylene/**4a** (incorp. = 6.0 mol%), and (**c**) ethylene/**4a** (incorp. = 1.8 mol%) copolymers.

The obtained copolymer is possibly used for producing plastic materials with improved adhesiveness and paintability rather than polyolefins with no incorporated functional groups. To show the improvement of wettability, the water contact angle was compared between E/IB and E/**4a** copolymers to evaluate the effect of hydroxy groups incorporated into the copolymer (Figure 6). A self-standing film is not obtained for these copolymers because the polymer yield is not enough, but thin films can successfully be fabricated on polyimide film by pressing the polymer at the melting condition (180 °C). Incorporating **4a** significantly decreased the water contact angle according to the incorporation ratio, which showed that hydroxy groups improved the hydrophilicity of the copolymer.

## 4. Conclusions

Various oxygen-containing vinylidenes were applied to copolymerization with ethylene using fluorenylamido-ligated titanium complexes in the presence of MMAO cocatalyst. The copolymerization of ethylene with 6-methyl-hept-6-en-1-ol derivatives (**4a**/**4b**) using catalyst **2a** produced copolymers with high molecular weight and incorporation ratios. The microstructure analysis using ^13^C NMR firmly established the isolated sequence structure of the incorporated comonomer. Isoprenol derivatives **5b** and plant-derived monomer **6b** were also successfully copolymerized, although the polymer yield was lower than that of ethylene/**4b** copolymer. Silyl groups in these comonomers can be deprotected by a standard method using organic fluoride. The obtained hydroxy-substituted copolymer film showed higher hydrophilic surface properties than those of the ethylene/isobutene copolymer.

Overall, we have introduced the first synthetic example of copolymers of ethylene and polar 1,1-disubstituted olefins using an early transition metal catalyst. Our findings also demonstrated an efficient strategy for applying various plant-derived monomers to functionalized polyolefin materials.

## Data Availability

Data are contained within the article and supplementary materials.

## Supplementary Materials

The following supporting information can be downloaded at: https://www.mdpi.com/article/10.3390/polym16020236/s1. Figures S1–S6: NMR spectra of new compounds; Figures S7–S13: NMR spectra of copolymers; Figure S14: GPC traces of copolymers; Figure S15: IR spectra of copolymers; Figure S16: DSC thermograms of copolymers.
