# Supplementary material for: Copolymerization of Ethylene with Functionalized 1,1-Disubstituted Olefins Using a Fluorenylamido-Ligated Titanium Catalyst
Source: Polymers (Basel). 2024 Jan 15;16(2):236. doi: 10.3390/polym16020236 (PMC10818569; doi:10.3390/polym16020236)
Supplement: Supplementary file 1 [file polymers-16-00236-s001.zip › polymers-2802455-supplementary.pdf]

Supporting Information

Copolymerization of Ethylene with functionalized 1,1-Disubstituted Olefins using a fluorenylamido-ligated titanium catalyst

Oluwaseyi Aderemi Ajala, Moeko Ono, Yuushou Nakayama, Ryo Tanaka, Takeshi Shiono*

Graduate School of Advanced Science and Engineering, Hiroshima University

1-4-1 Kagamiyama, Higashi-hiroshima, 739-8527 Japan.

Table of Contents

NMR spectra of new compounds	2
NMR spectra of copolymers	8
GPC traces of polymers	12
IR spectra of polymers	13
Thermal properties of polymers	14

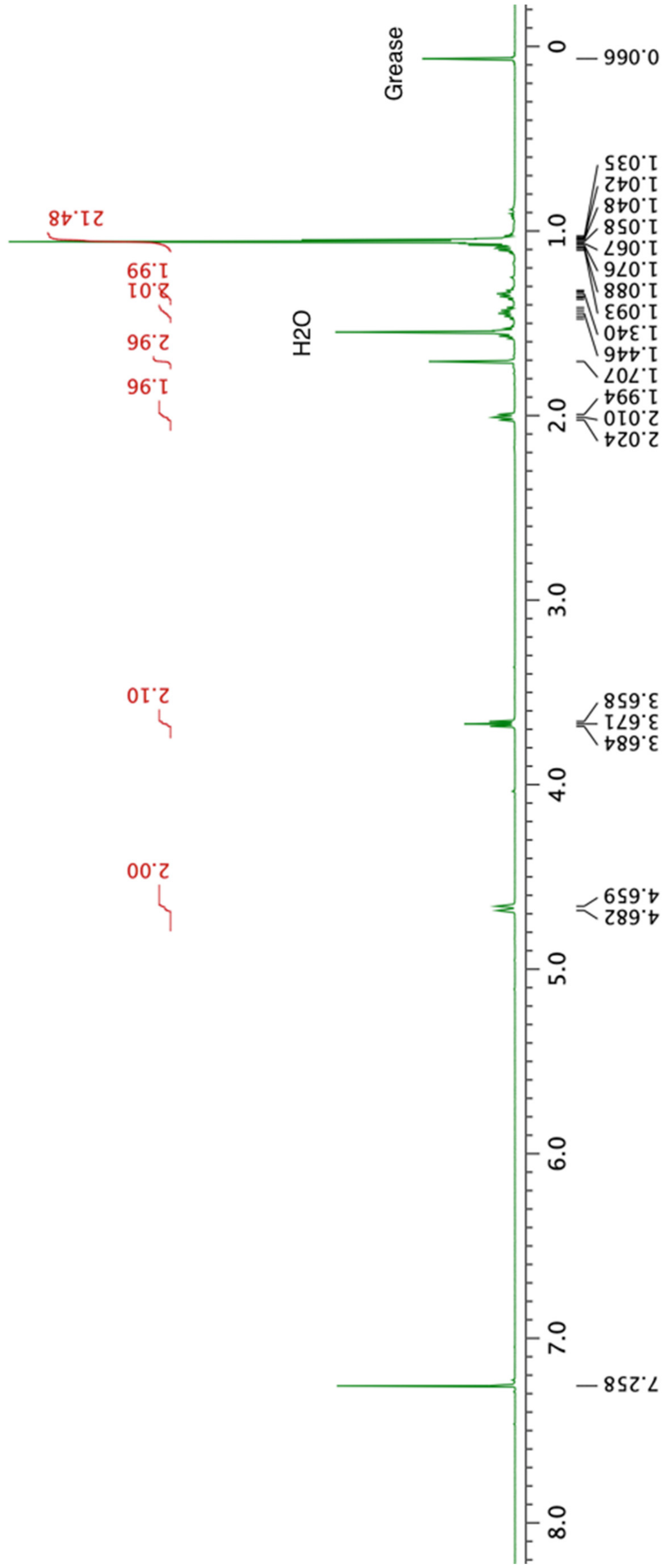

Figure S1. ^{1}H NMR spectrum of compound **4b** (500 MHz, in $CDCl_3$).

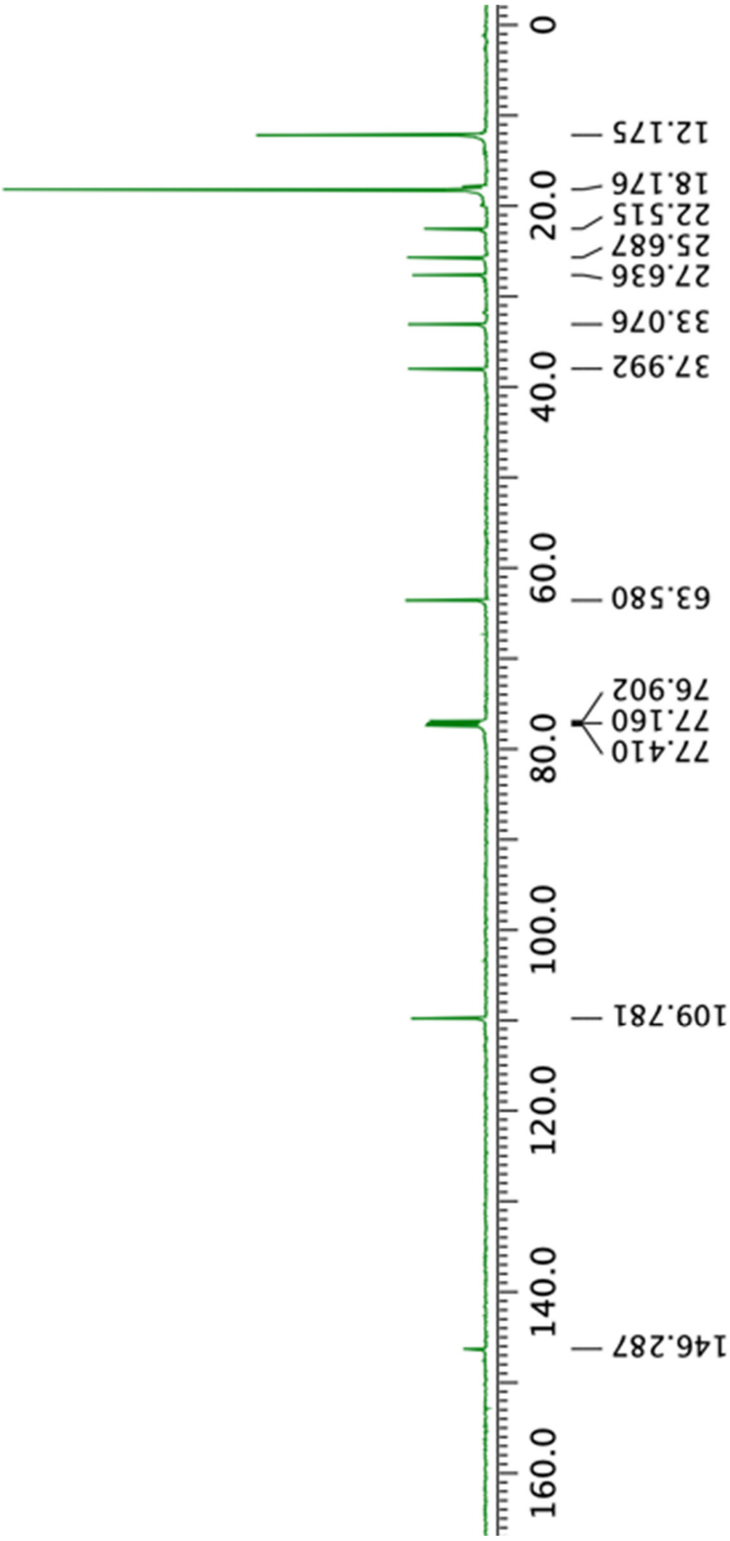

Figure S2. ^{13}C NMR spectrum of compound **4b** (125 MHz, in $CDCl_3$).

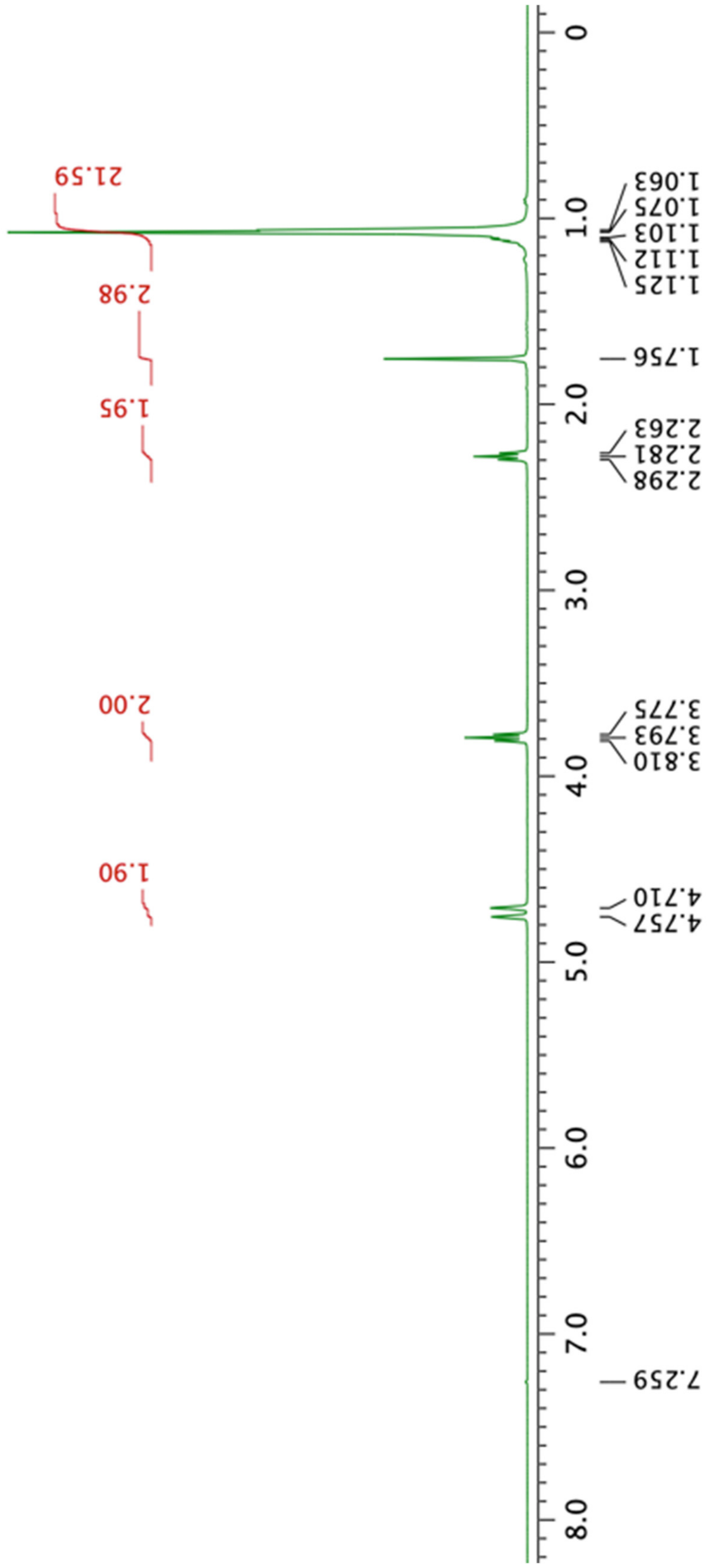

Figure S3. ^{1}H NMR spectrum of compound **5b** (400 MHz, in $CDCl_3$).

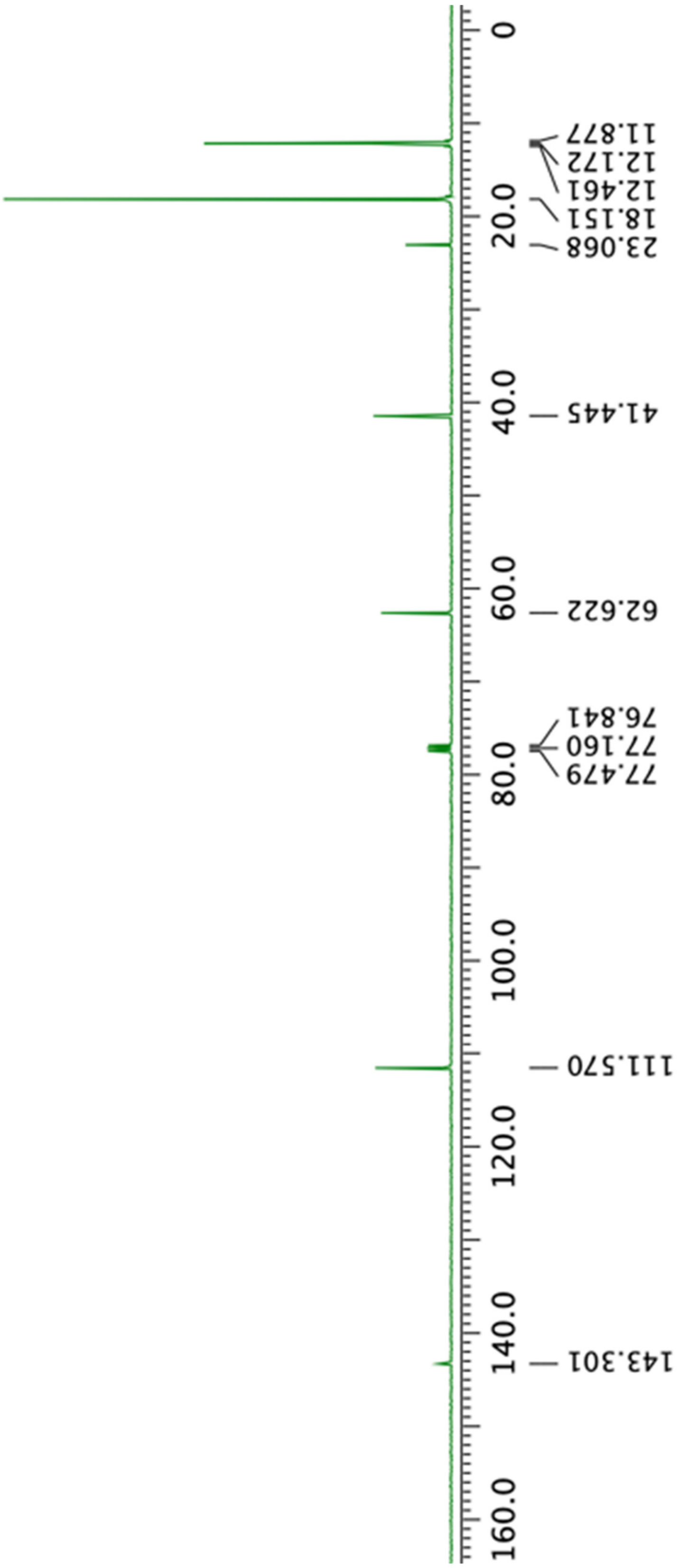

Figure S4. ^{13}C NMR spectrum of compound **5b** (100 MHz, in $CDCl_3$).

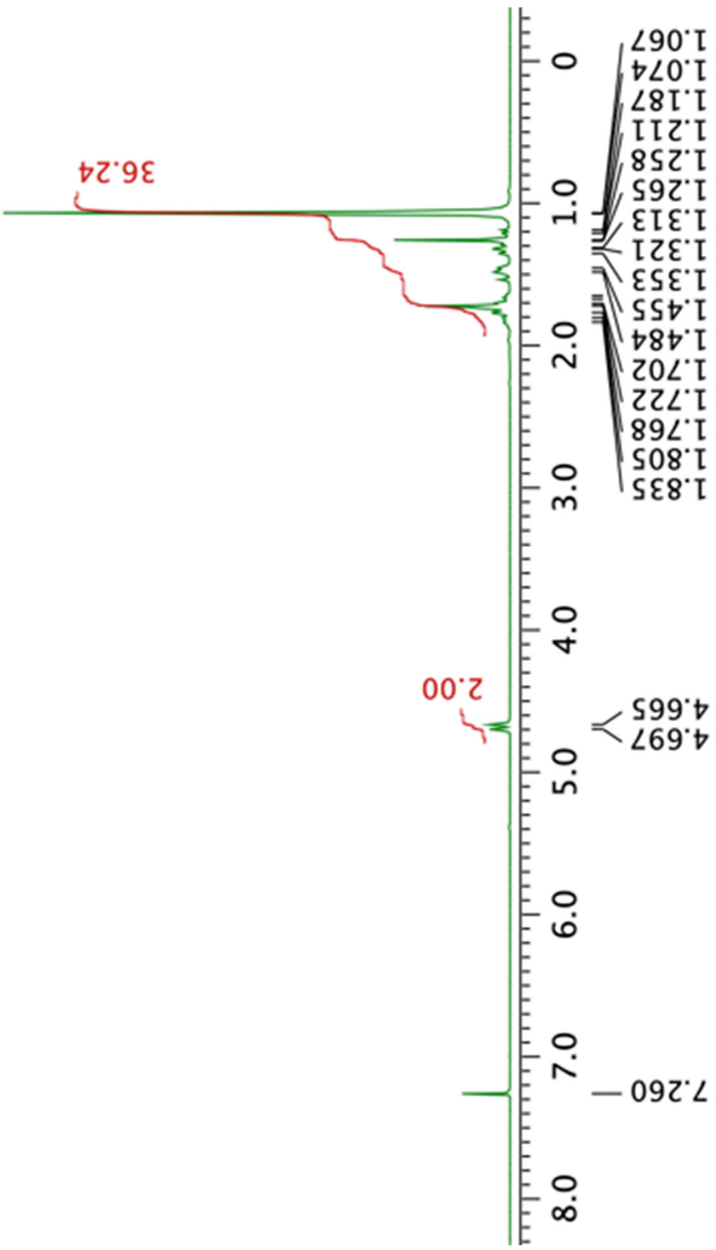

Figure S5. ^{1}H NMR spectrum of compound **6b** (400 MHz, in $CDCl_3$).

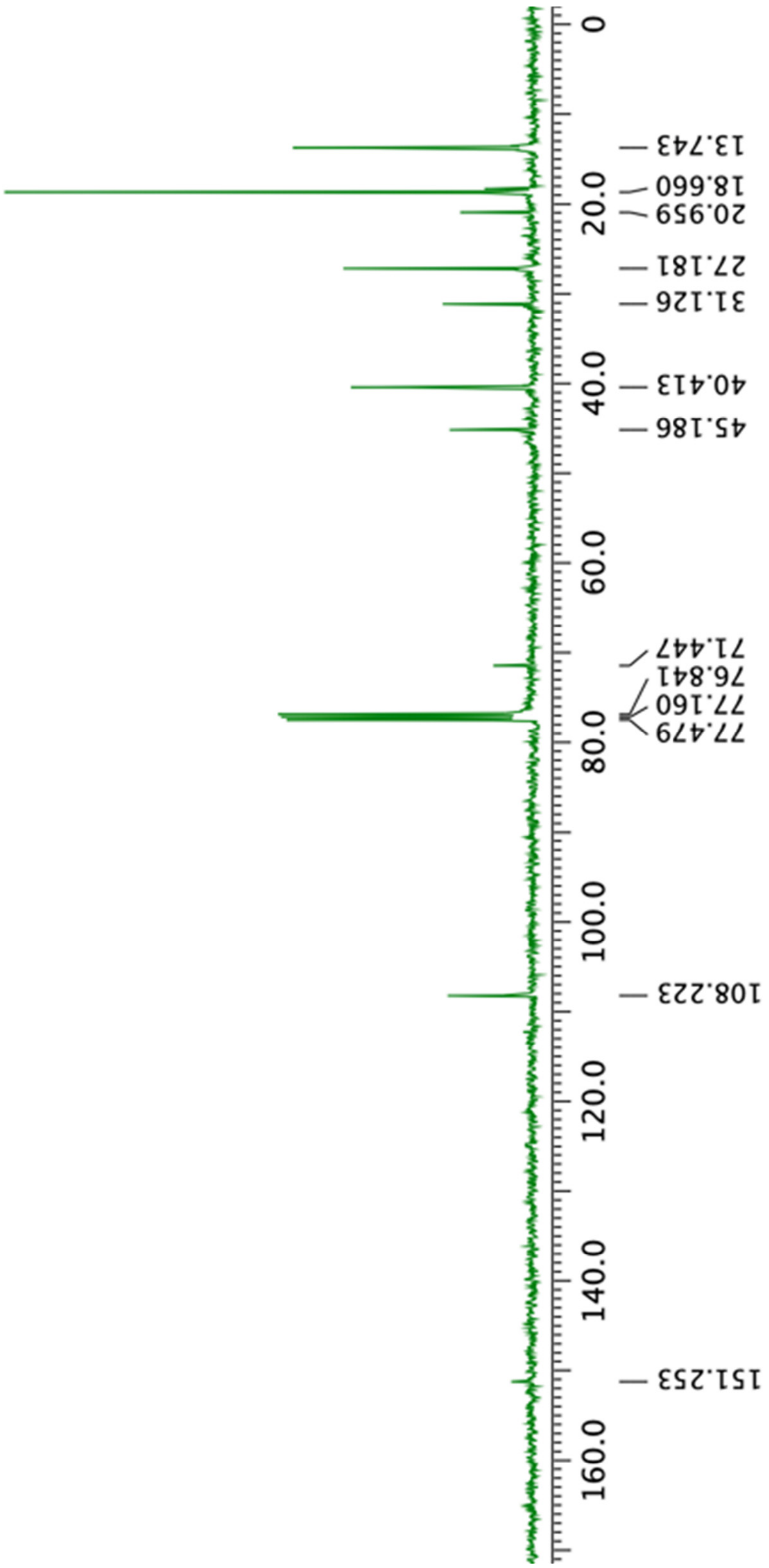

Figure S6. ^{13}C NMR spectrum of compound **6b** (100 MHz, in $CDCl_3$).

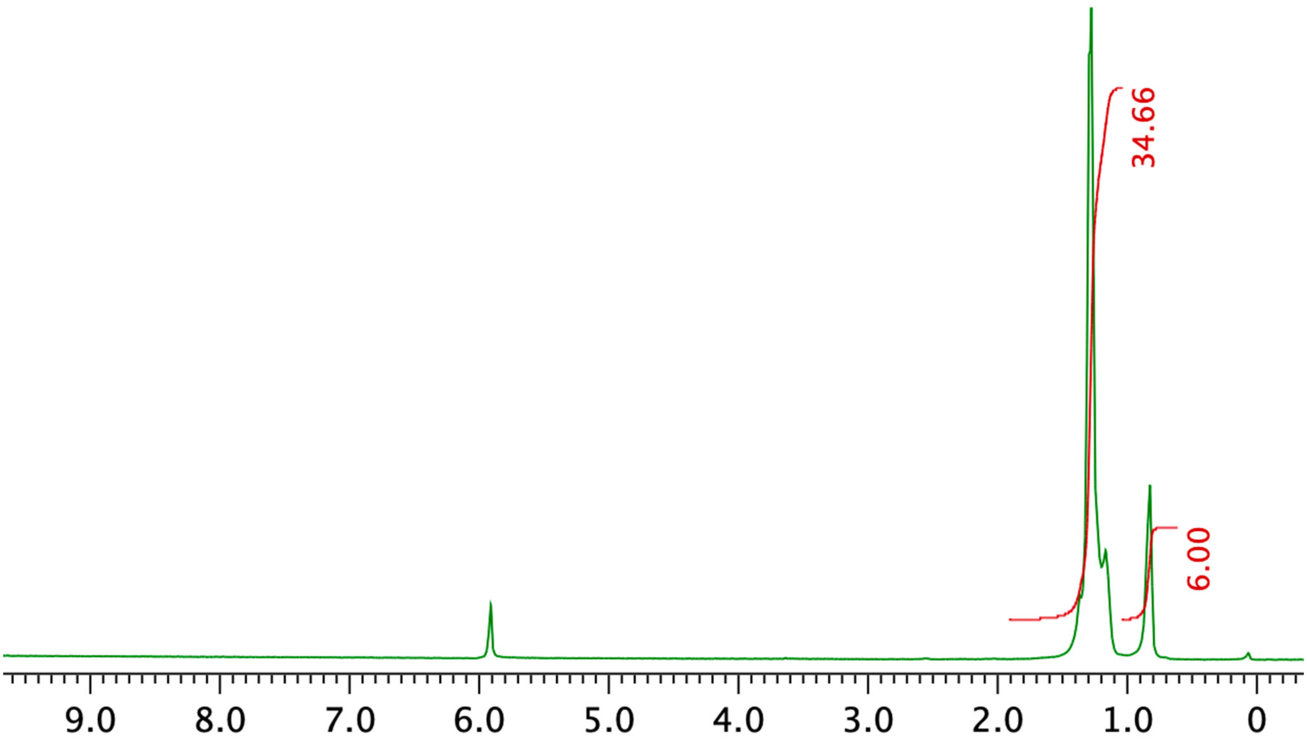

Figure S7. ^{1}H NMR spectrum of ethylene/isobutene copolymer (Table 1, run 1, 500 MHz, in $C_2D_2Cl_4$).

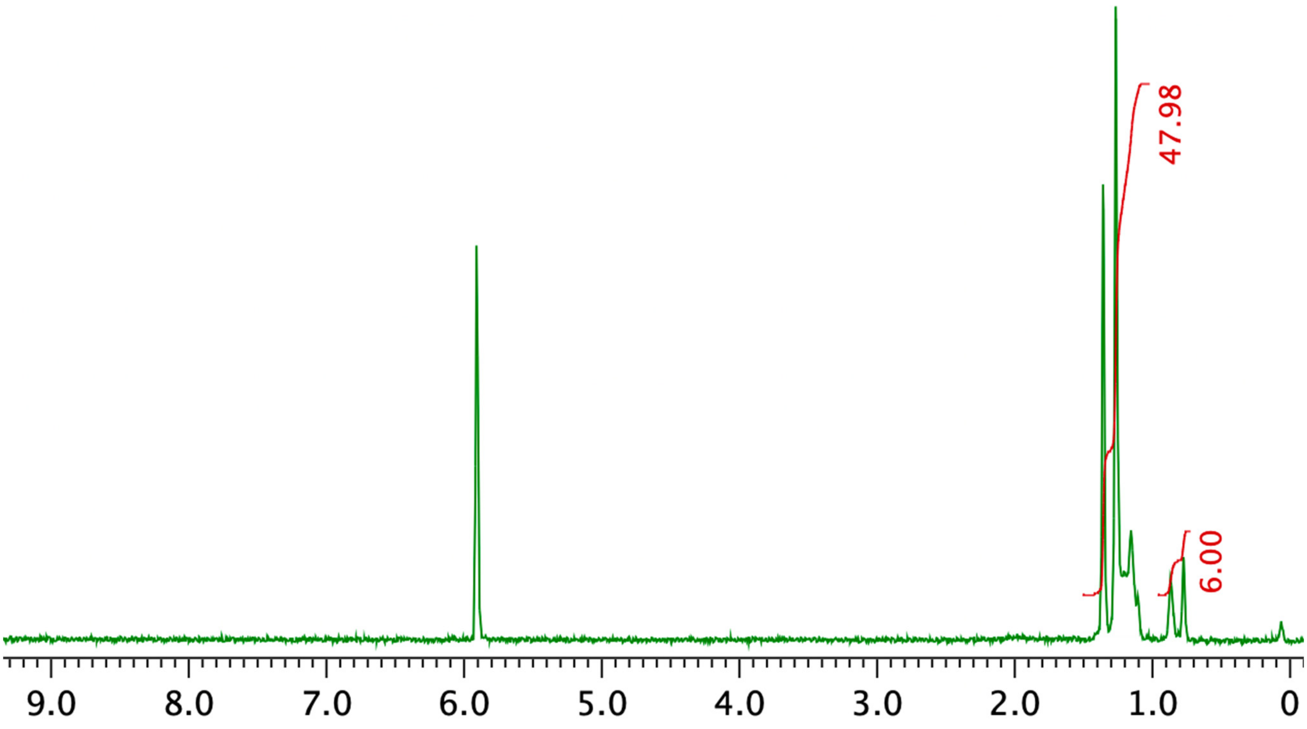

Figure S8. ^{1}H NMR spectrum of ethylene/2M1P copolymer (Table 1, run 2, 500 MHz, in $C_2D_2Cl_4$).

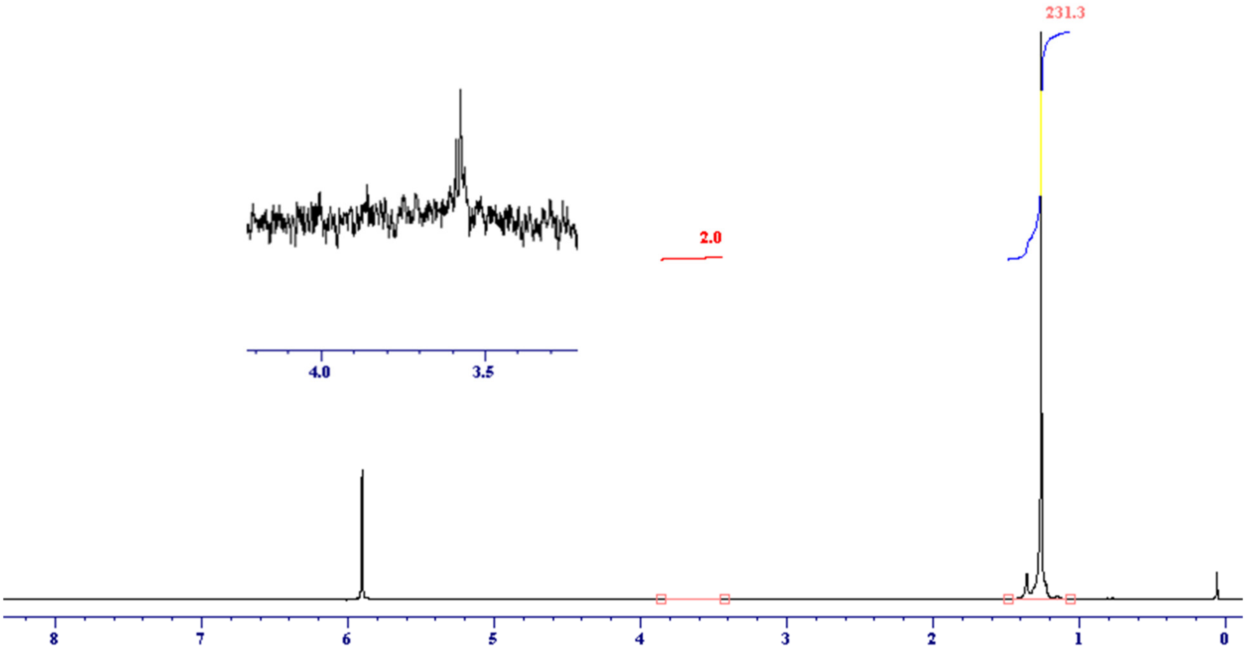

Figure S9. ^{1}H NMR spectrum of ethylene/**4a** copolymer (Table 1, run 4, 500 MHz, in $C_2D_2Cl_4$).

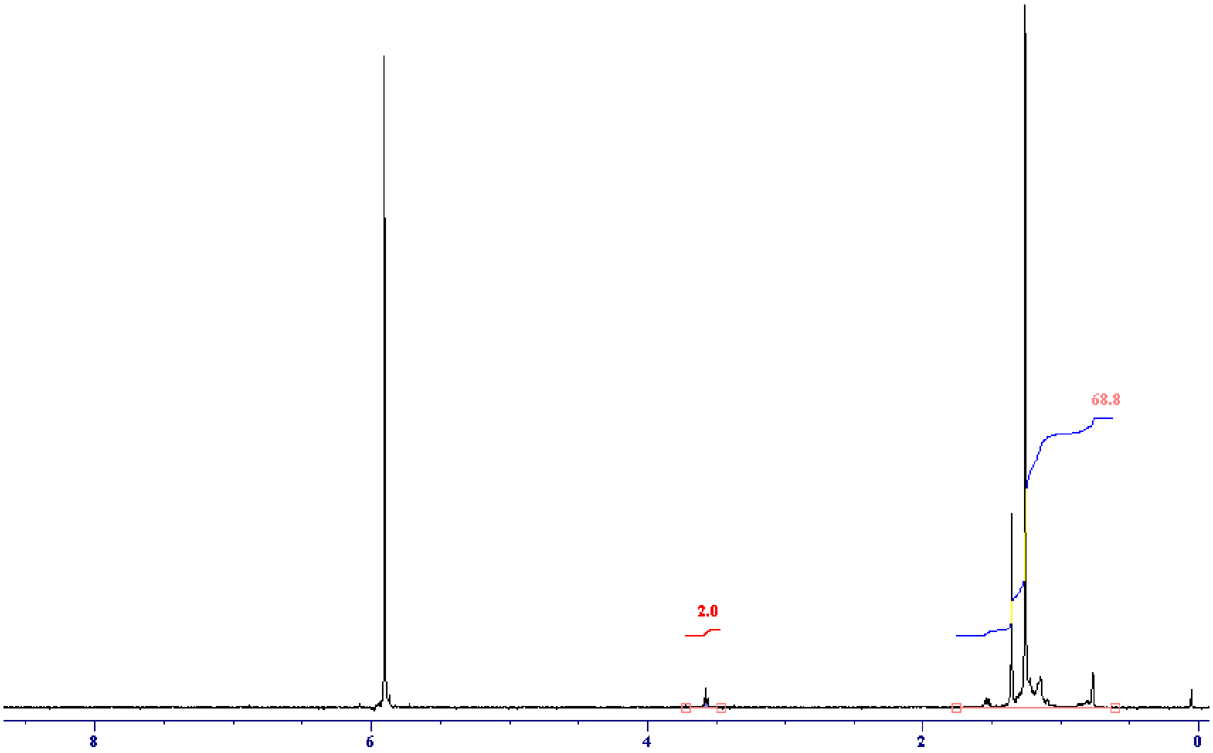

Figure S10. ^{1}H NMR spectrum of ethylene/**4b** copolymer after deprotection (Table 1 , run 3, 500 MHz, in $C_2D_2Cl_4$).

Run 5

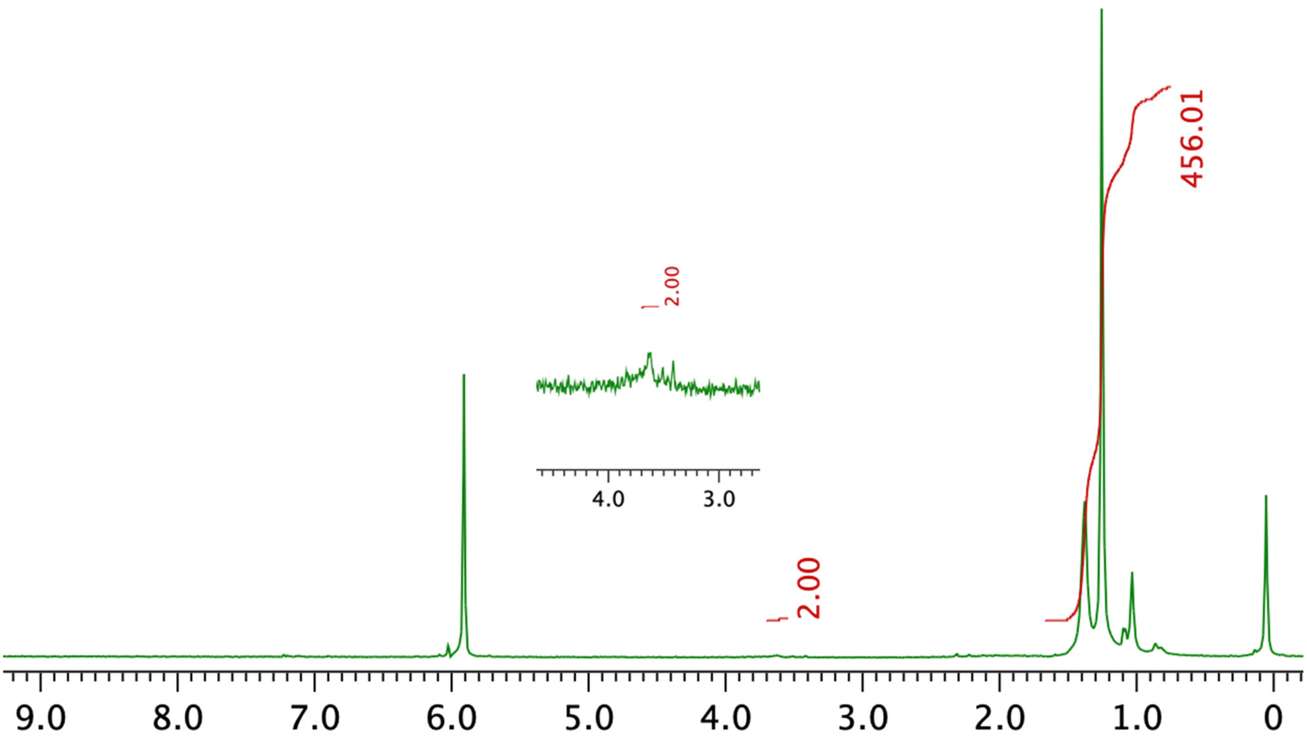
456.01
2.00
2.00
4.0
3.0
9.0
8.0
7.0
6.0
5.0
4.0
3.0
2.0
1.0
0

Run 6

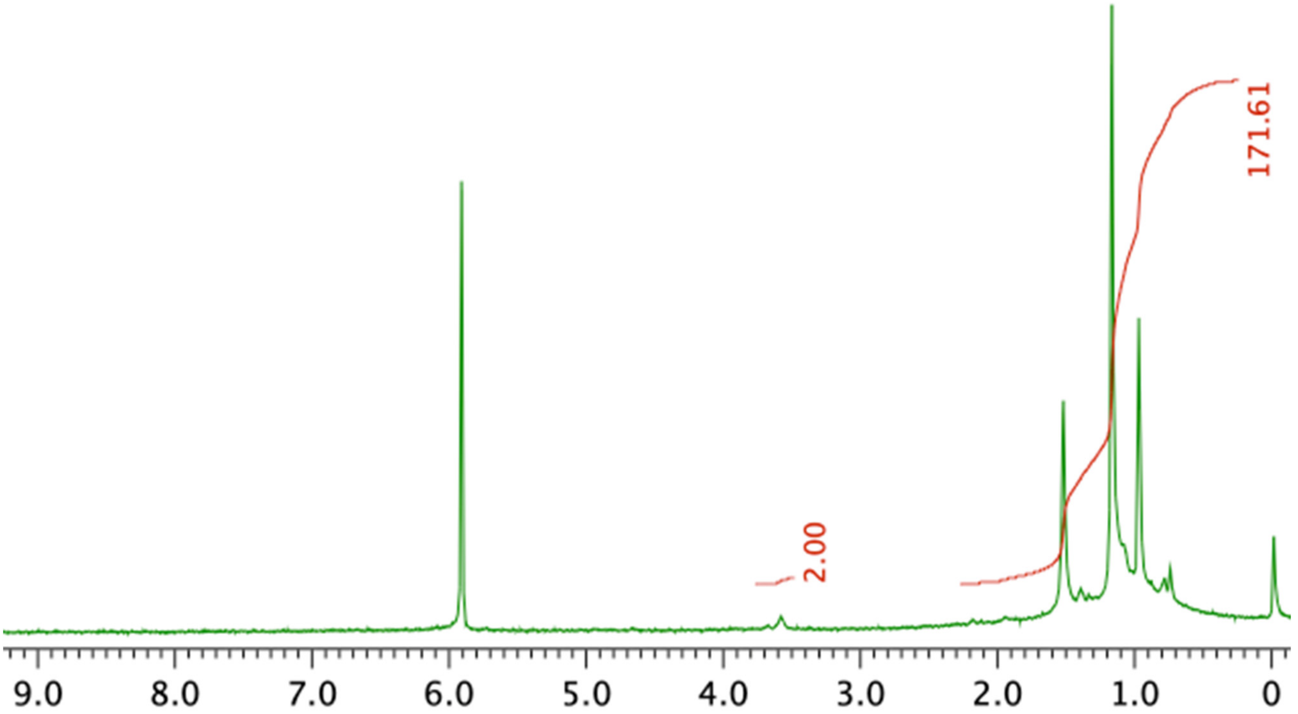
171.61
2.00
9.0
8.0
7.0
6.0
5.0
4.0
3.0
2.0
1.0
0

Run8

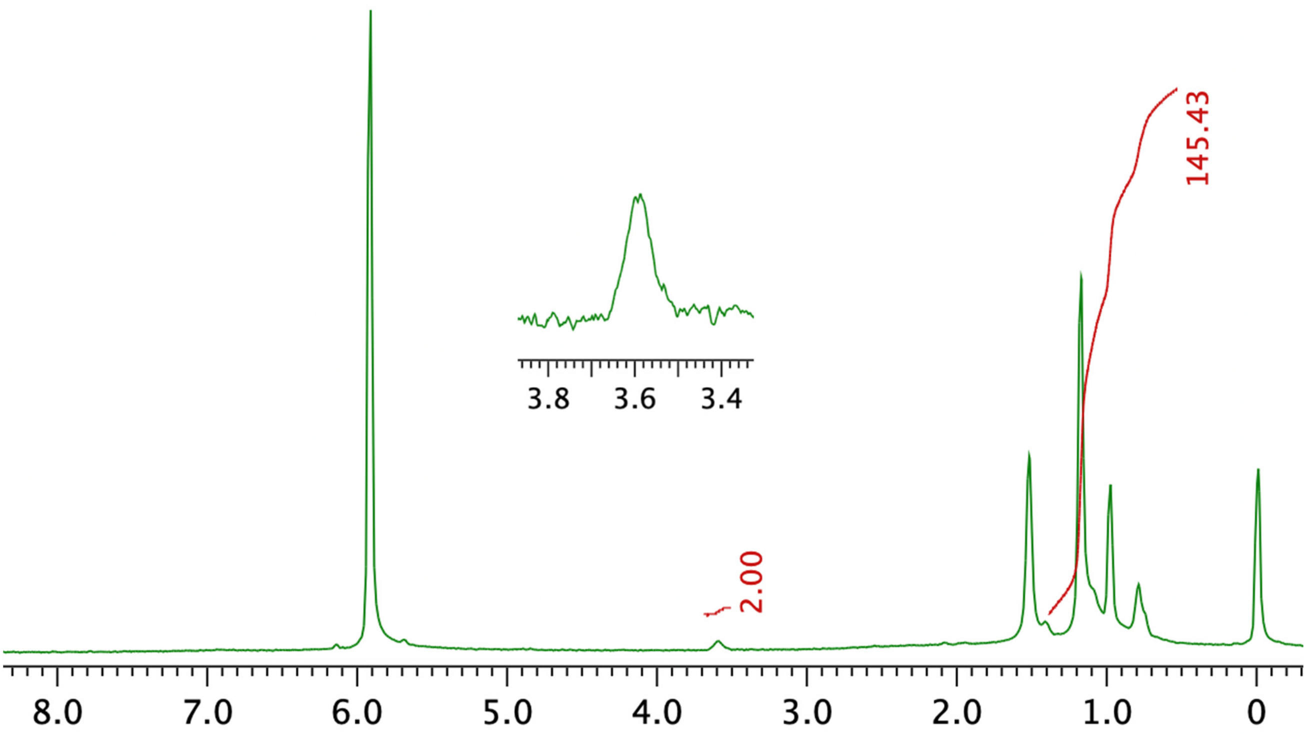

Run9

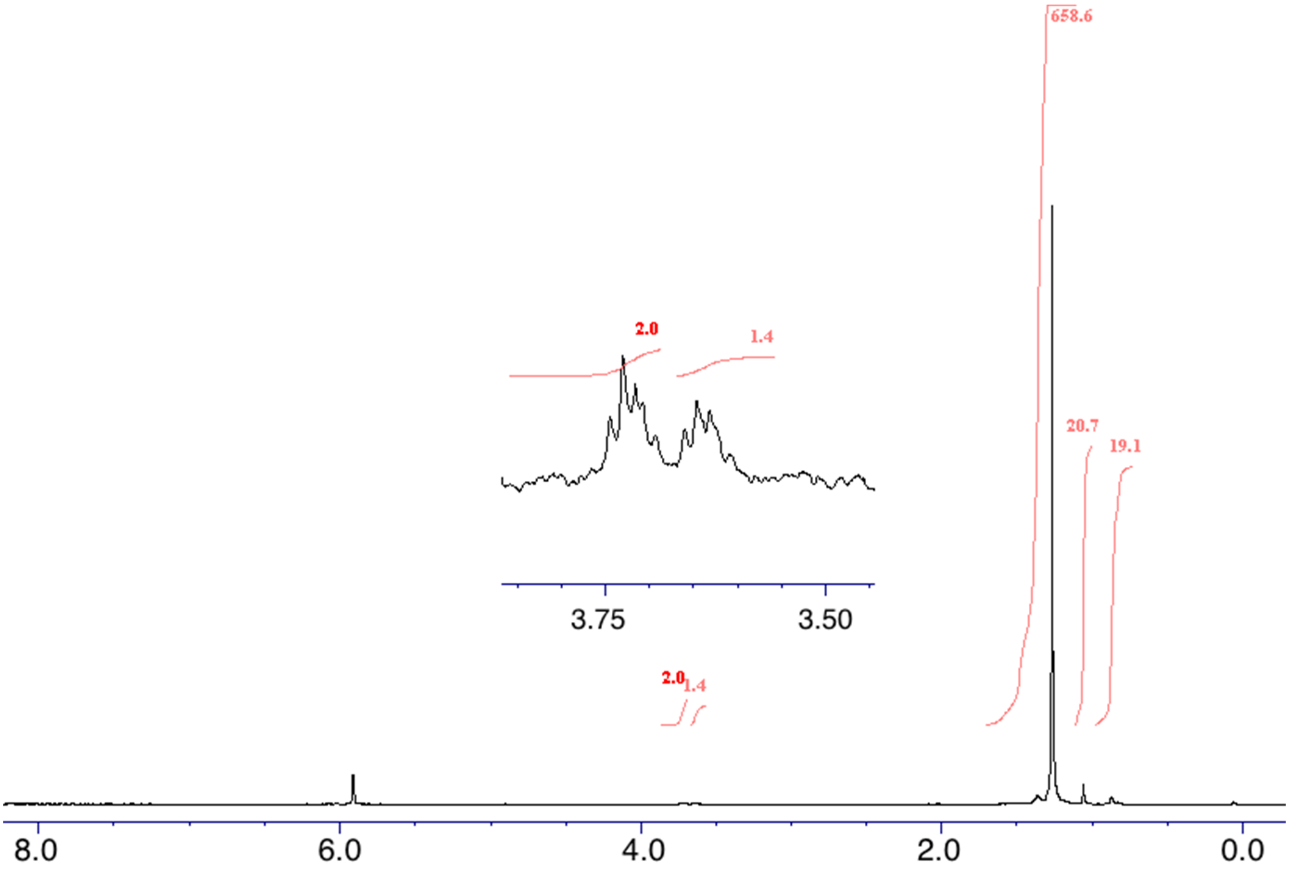

Figure S11. ^{1}H NMR spectra of ethylene/**5b** copolymers (500 MHz, in $C_2D_2Cl_4$, 130 °C).

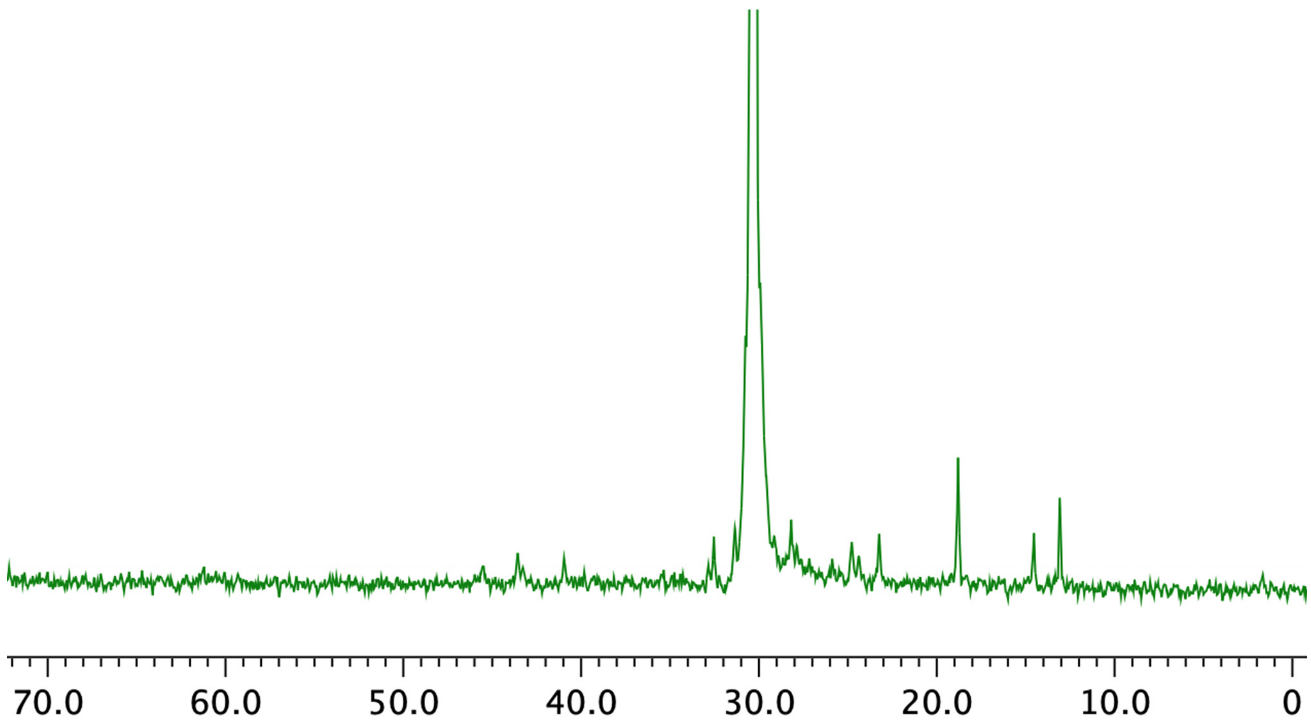

Figure S12. ^{13}C NMR spectrum of ethylene/**5b** copolymer
(Table 1, Run 6, 125 MHz, in 1,1,2,2,-tetrachloroethane-d_2, 130 °C).

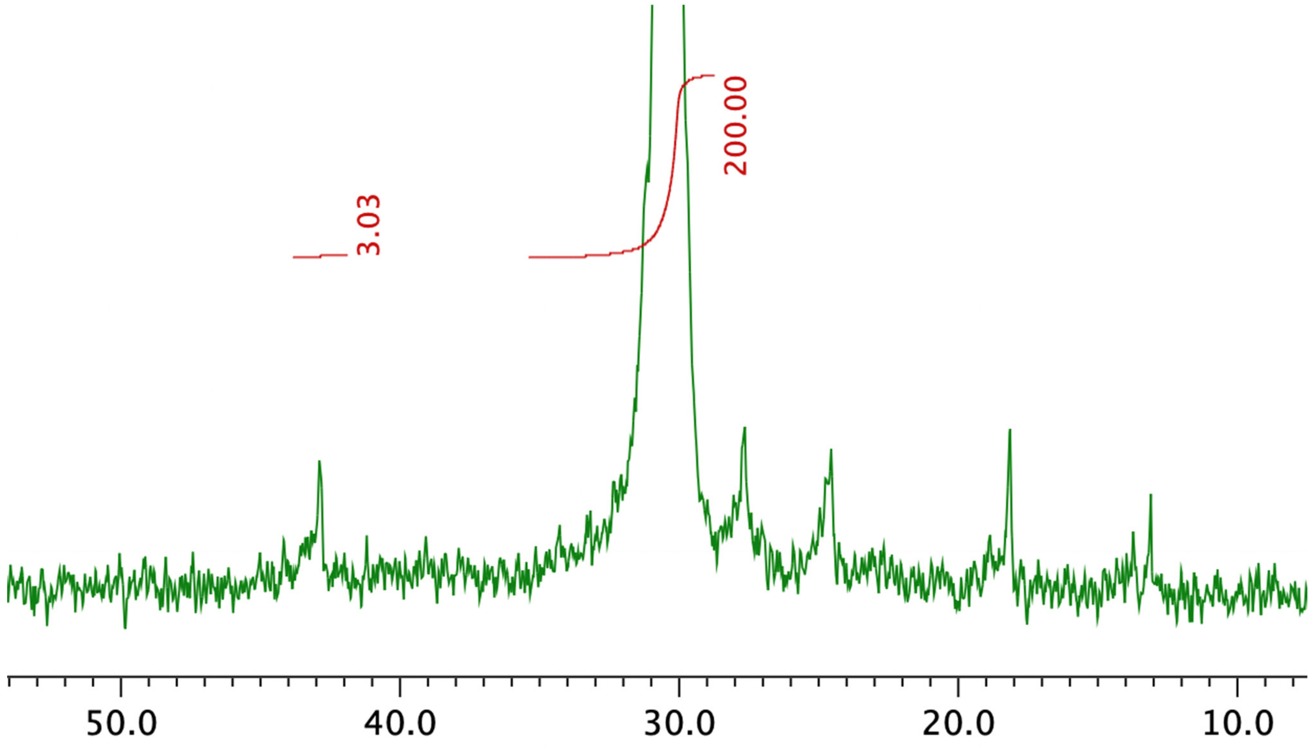

Figure S13. An enlarged view of ^{13}C NMR spectra of ethylene/**6b** copolymer (Scheme 4, 125 MHz, in 1,1,2,2,-tetrachloroethane-d_2, 130 °C).

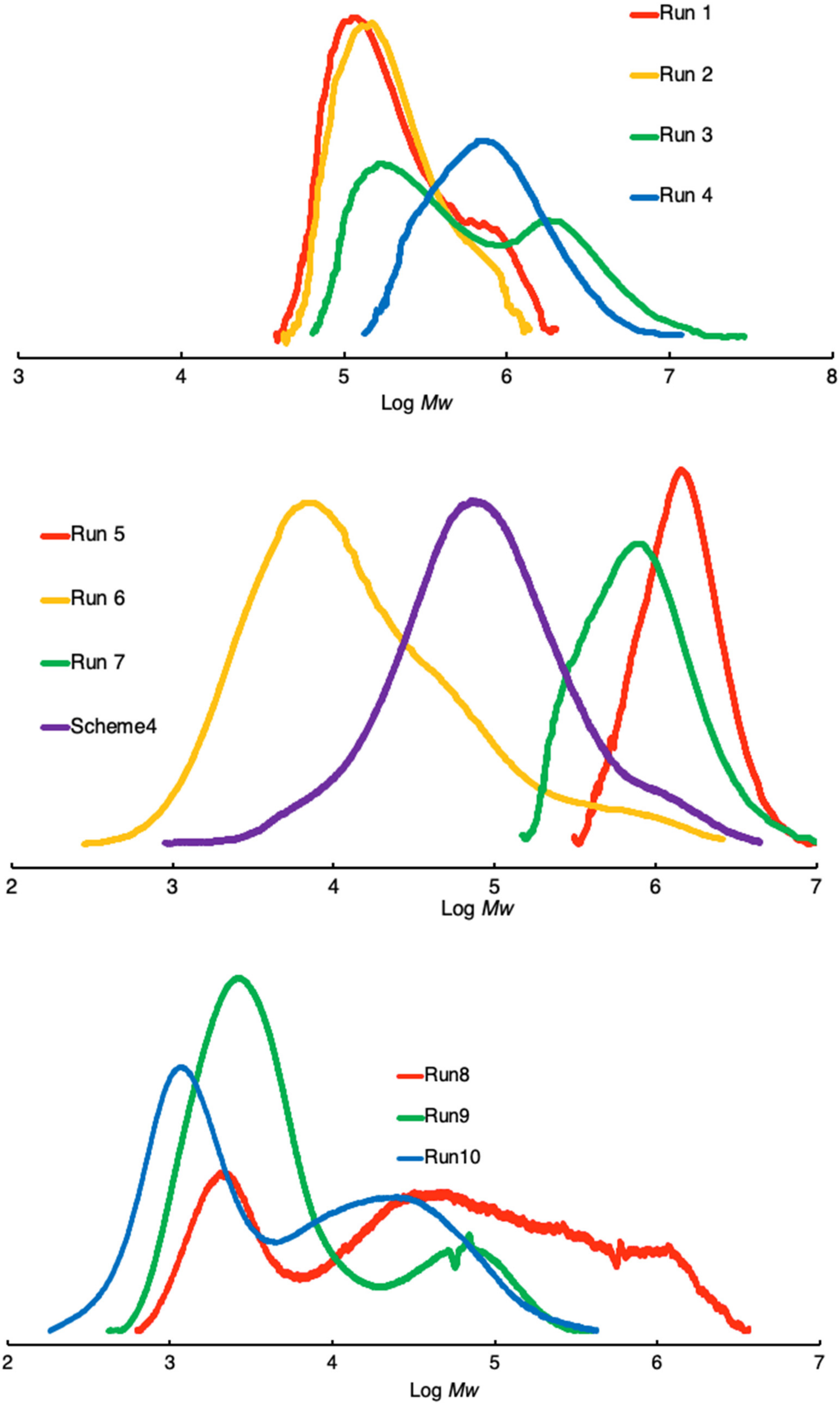

Figure S14. GPC traces of copolymers.

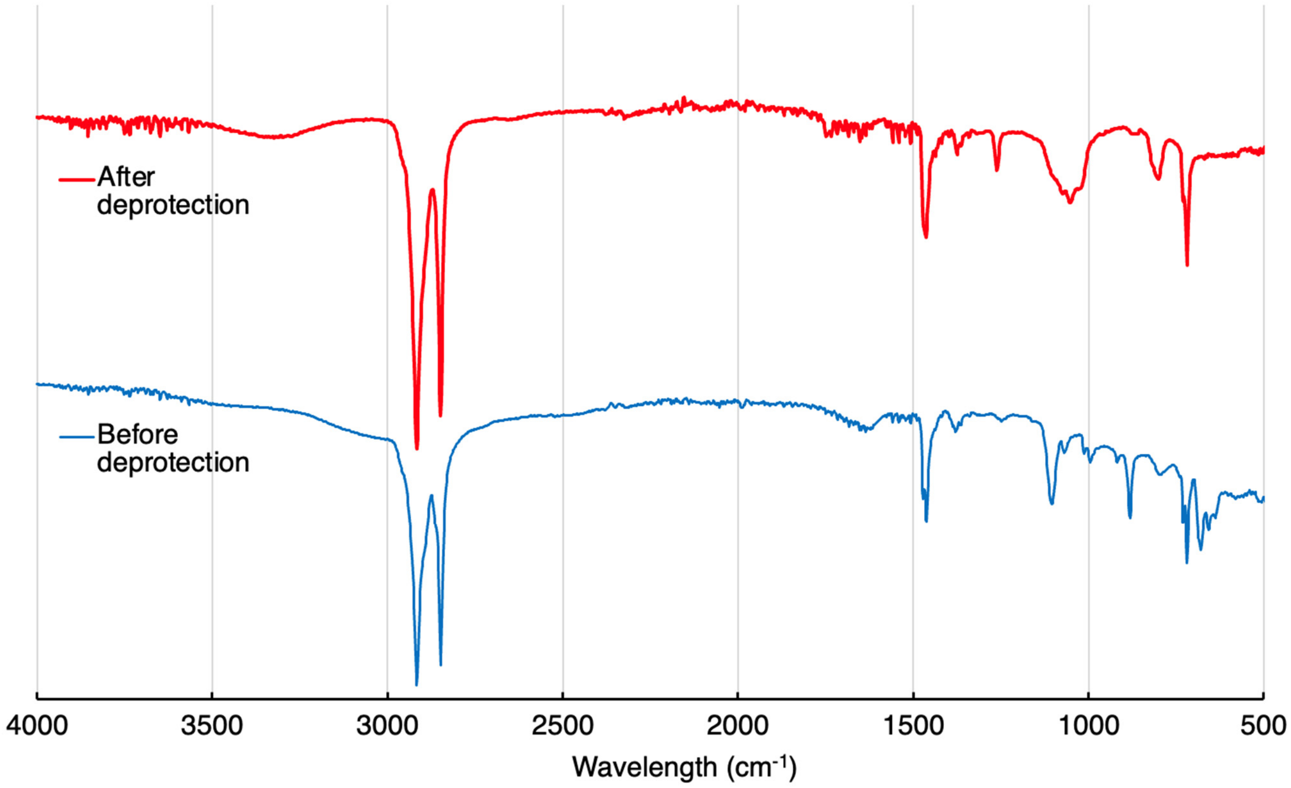

Figure S15. IR spectrum of ethylene/**4b** copolymer obtained in Table 1, run 3, before and after deprotection.

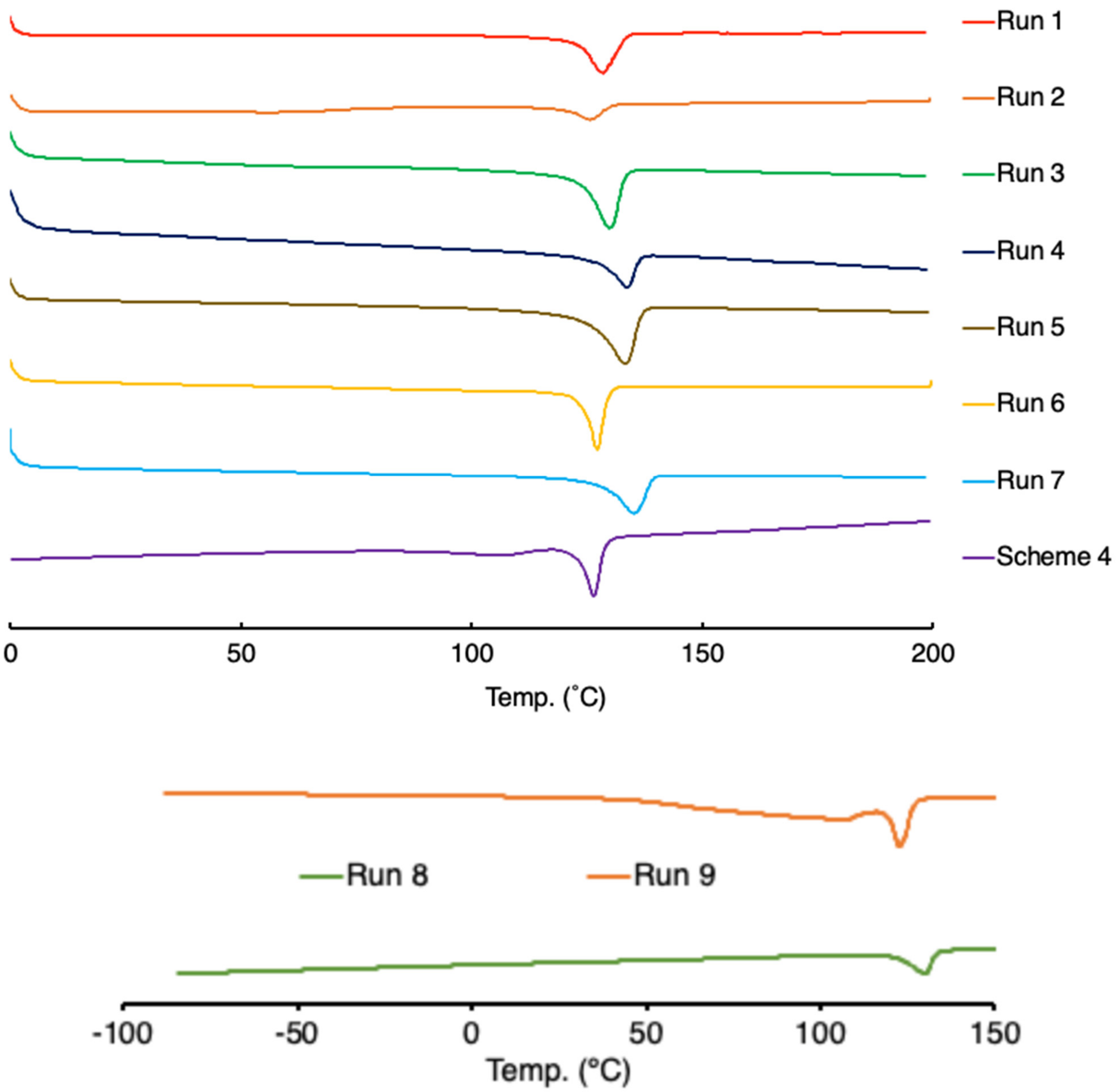

Figure S16. DSC thermograms of copolymers.
